# Sun Protection as a Strategy for Managing Heat Stress in Avocado Trees

**DOI:** 10.3390/plants13202854

**Published:** 2024-10-11

**Authors:** Francisco José Domingues Neto, Débora Cavalcante dos Santos Carneiro, Marcelo de Souza Silva, Marco Antonio Tecchio, Sarita Leonel, Adilson Pimentel Junior, Elizabeth Orika Ono, João Domingos Rodrigues

**Affiliations:** 1 School of Agriculture Sciences, Sao Paulo State University (UNESP), Botucatu, Sao Paulo 18610-034, Brazilmarcelo.s.silva@unesp.br (M.d.S.S.); marco.a.tecchio@unesp.br (M.A.T.);; 2 Institute of Biosciences, Sao Paulo State University (UNESP), Botucatu, Sao Paulo 18610-034, Braziljoao.domingos@unesp.br (J.D.R.)

**Keywords:** heat stress, avocado, *Persea americana*, sun protection, kaolin, titanium dioxide, calcium oxide, photosynthesis, fruit quality

## Abstract

The increasing incidence of heat stress due to global climate change poses a significant challenge to avocado (*Persea americana*) cultivation, particularly in regions with intense solar radiation. This review evaluates sun protection strategies, focusing on the efficacy of different sunscreen products such as kaolin, titanium dioxide, and calcium oxide in mitigating thermal stress in avocado trees. The application of these materials was shown to reduce leaf and fruit surface temperatures, improve photosynthetic efficiency, and enhance fruit quality by preventing sunburn and dehydration. Despite these benefits, challenges remain, including the optimal timing and dosage of application, and the potential residue impacts on fruit marketability. The review emphasizes the need for ongoing research to develop more effective formulations and to integrate these sun protection strategies with other agronomic practices. The role of extension services in educating producers about the proper use of these technologies is also highlighted as crucial for the successful adoption of sun protection measures in avocado farming.

## 1. Introduction

Brazil is one of the world’s leading fruit producers, distinguished by its vast territorial extension and privileged geographic location, which allow for the cultivation of a wide variety of fruit species, from tropical to temperate climate origins [1]. Fruit farming plays a crucial role in Brazilian agribusiness, generating income, creating jobs, and significantly contributing to food security both nationally and internationally [2]. As such, fruit production is distributed across all states in the federation, generating thousands of jobs and promoting regional development. In 2023, Brazilian fruit production surpassed 42 million tons, covering around 2.6 million hectares, equivalent to 0.3% of the national territory. In this context, family farming plays a prominent role, accounting for 81% of the total rural properties in Brazil (940,000 properties). The fruit sector is also notable for its high employment rate, generating more than 195,000 formal jobs, which represents 12% of all jobs in Brazilian agriculture [3].

The state of São Paulo, in particular, stands out as the largest fruit producer in the country, with 18.11 million tons (34.76% of national production), with at least 635 of its 645 municipalities having an Agricultural Production Unit (UPA) engaged in fruit farming [3].

Regarding avocado production, Brazil is one of the world’s leading producers, with significant production concentrated in key states such as São Paulo, which leads national avocado production, followed by states like Minas Gerais and Paraná. In 2023, Brazilian avocado production was estimated at around 300,000 tons, cultivated over approximately 15,000 hectares. The state of São Paulo, in particular, contributes a significant share of this production, accounting for over 50% of the national total. This production is predominantly intended for the domestic market, although exports are growing, especially to European countries, Chile, and Costa Rica. Additionally, India, the USA, and Japan are promising markets for Brazilian avocados [4].

However, avocado production in Brazil faces significant challenges, particularly concerning environmental stresses, among which thermal stress caused by intense solar radiation and increasing global temperatures are the most concerning. The intensification of climate change has exacerbated these problems, negatively impacting plant development, fruit production, and quality [5,6]. Changes in seasonal patterns of rainfall and, especially, temperature are even more impactful for perennial fruit crops like avocado trees, which may have their ideal growing conditions shifted to other latitudes where soil characteristics may not be the most favorable, or which may favor the emergence of pests and diseases in current growing regions [7,8].

These climate changes may further interfere with the duration of the plant cycle, hormonal and nutritional balance, and the quantity and quality of production, directly and indirectly affecting the sustainability of avocado production in the main producing regions [8,9,10]. In this context, the implementation of sun protection techniques becomes an essential strategy for managing thermal stress in fruit trees. The use of suitable sunscreens, such as kaolin, films, shading screens, reflective substances, and other innovative methods, can reduce the intensity of direct solar radiation, providing a more moderate environment for plants, especially those with higher sensitivity to excessive solar radiation.

The avocado tree, for example, is particularly sensitive to thermal stress, with damage caused by excessive temperatures and ultraviolet (UV) light, which can result in burns on leaves and fruits, significantly compromising productivity and the commercial quality of harvested products [11,12]. Such damage not only compromises yield but also affects the economic viability of cultivation, especially in areas intended for export. High temperatures during the flowering of avocado trees, combined with water stress and low air humidity, can have a significant negative effect on fruit sets, resulting in lower fruit production and directly affecting productivity [13,14]. Therefore, sun protection measures and appropriate thermal stress management are essential to ensure healthy and stable avocado production.

To mitigate these adverse effects, the use of sunscreens has emerged as a promising strategy for managing thermal stress in fruit trees. These products, such as kaolin, are applied directly to leaves and fruits, forming a physical barrier that reduces the intensity of solar radiation, minimizing overheating and associated damage. Studies have shown that the use of kaolin can reduce leaf and fruit temperature, improve the reflectance of these structures, and decrease transpiration, resulting in improved fruit quality and productivity [15].

In addition to kaolin, other products have been investigated for sun protection in fruit trees, including titanium dioxide, calcium oxide, and various films and shading screens. These materials also act as physical barriers that reflect solar radiation and, in some cases, as with the use of titanium dioxide, can provide additional protection against UV rays. Kaolin, in particular, a mineral composed of aluminum silicate (Al4 Si4 O10 [OH]8), characterized by its fine particles, white color, flat shape, and porosity, stands out for its easy dispersion properties, low toxicity, and non-reactivity over a wide pH range, making it one of the most used and researched sunscreens. However, selecting the most appropriate product should consider the fruit species, specific environmental conditions, and ease of application and removal of the product after harvest.

Despite promising results, the use of sunscreens on avocado trees is still in the early stages of research, and their effectiveness may vary depending on environmental conditions and the formulation of the product used. In this sense, it is crucial to develop strategies that integrate scientific research with agricultural practice to ensure the success of these techniques and their proper implementation by fruit growers throughout the country.

The objective of this literature review is to compile and analyze the main sun protection strategies used to mitigate the effects of thermal stress on fruit trees, with an emphasis on the avocado tree (*Persea americana* Mill). Specifically, the review aims to evaluate the effectiveness of different sunscreens, such as kaolin, titanium dioxide, calcium oxide, and other innovative methods, in reducing damage caused by intense solar radiation, improving fruit quality, and increasing crop productivity.

## 2. Methodology

This literature review was conducted through a comprehensive analysis of the available scientific literature on sun protection strategies for fruit trees, with a specific focus on studies involving the use of kaolin and other products to mitigate thermal stress in avocado trees. The literature search included scientific articles, theses, dissertations, technical reports, and publications from government agencies and research institutes. The databases used for the search were Scopus, Web of Science, Google Scholar, and SciELO.

The inclusion criteria were studies that evaluated the effectiveness of sunscreens in fruit trees; studies that addressed the impact of thermal stress on fruit trees and the damage caused by solar radiation; research that compared different sun protection products; and relevant publications on agronomic practices for mitigating thermal stress in avocado trees.

The data extracted from the selected studies included type of sunscreen used, application method, concentrations, environmental conditions of the experiments, impacts on plant physiology and fruit quality, and practical recommendations for growers.

## 3. Development

### 3.1. Importance of Brazilian Fruit Farming

Brazil is one of the world’s leading fruit producers, with significant growth in fruit production driven by the country’s vast territorial extension and favorable geographical location [1,16]. Currently, Brazil ranks third in fruit production volume, with approximately 59 million tons, behind only China and India [4]. This activity is crucial for agribusiness, generating income and employment while supplying both domestic and international markets with quality products [2].

Furthermore, the Brazilian fruit sector has made strides in sustainable practices and technological innovations, such as precision irrigation and integrated pest management. These advancements promote more efficient production while reducing environmental impact. Initiatives to certify good agricultural practices are becoming progressively common, aligning fruit production with global demands for sustainable food [17,18,19].

Avocado has gained particular prominence among cultivated fruits due to its versatility and nutritional value. Its production benefits from favorable climatic conditions across various regions of Brazil, significantly contributing to both the domestic market and export sales. Alongside other fruits like mango, grapes, and bananas, avocados have become attractive options for demanding international markets, reflected in the increasing global interest in Brazilian exports.

It is a fruit well known for its high content of bioactive compounds. Due to its health benefits, its consumption has increased globally in recent years, with demand outstripping the volume produced [20]. In 2022, the world production of avocados was 8.6 million tons [4]. Among the producing countries, Mexico stands out with the largest world production, followed by Colombia, Peru, Dominican Republic, Kenya and Brazil, respectively. Currently, Brazil exports a small volume of this fruit in comparison to other countries such as Colombia, Peru, Chile, and Mexico. The opening of new avocado buyers in countries on the American and Asian continents is an additional attraction for production in Brazil [2,4].

In relation to the domestic market, the transformation was significant, driven by a number of factors. The growing domestic demand for this nutritious fruit is one of the main influencing factors. Changing food preferences and increased health consciousness among consumers have led to an increase in the consumption of avocados, which are rich in essential nutrients and antioxidants [20].

### 3.2. Plant Thermal Stress

#### 3.2.1. The Influence of Thermal Stress on Plants

Climate change, characterized by global temperature increases and the intensification of extreme events, has exacerbated problems related to thermal stress in fruit trees [21,22]. Fruit farming is severely impacted by extremely high temperatures, significantly affecting plant development processes from seed germination to vegetative growth and the reproductive phase [23].

Thermal stress affects basic physiological processes in plants, such as photosynthesis and respiration rates, stomatal conductance, and leaf water potential homeostasis [24]. Moreover, these climatic conditions, frequently observed in fruit cultivation areas, can interfere with net carbon assimilation by intensifying plant photorespiration rates, increasing mitochondrial respiration, inactivating the Rubisco enzyme, and reducing photosystem II activity. This can also interfere with stomatal conductance, depending on the plant species and the intensity of the abiotic stress [25,26].

This scenario challenges producers to adapt their agricultural practices to mitigate the negative impacts on fruit production and quality. The increased frequency and intensity of heatwaves, combined with prolonged drought periods, jeopardize the economic viability of various fruit crops, especially those of temperate and subtropical climates grown in regions where climatic conditions are already marginally suitable for cultivation. Adapting to these new climatic conditions requires an integrated approach, combining the use of sunscreens, improvements in water management, and the development of more heat-resistant cultivars [5,6].

Thermal stress resulting from excessive solar radiation can cause several adverse impacts on fruit trees. High temperatures can lead to direct damage, such as leaf burns, reduced photosynthesis rates, plant dehydration, and increased water loss through transpiration [27,28]. Additionally, thermal stress can negatively affect fruit development and quality, leading to reduced yields and changes in texture, color, flavor, and nutritional composition [29]. Plants subjected to these adverse conditions often exhibit visible symptoms, such as chlorotic, wilted, and necrotic leaves, compromising the crop’s health and economic viability. These direct damages may be associated with increased thylakoid membrane fluidity, the production of reactive oxygen species (ROS), loss of membrane integrity, and inhibition of protein regulation, which trigger lesions in different plant tissues [24,25]. These damages are proportional to the intensity and duration of exposure to high temperatures [26].

#### 3.2.2. The Influence of Thermal Stress on Avocado

The avocado tree is a fruit tree native to subtropical climates. However, even when grown in these conditions, the damage caused by abiotic stresses to plants was frequent, exacerbated by the lack of appropriate agronomic practices to protect the plants throughout the vegetative and reproductive development phases. This situation compromises the economic viability of avocado cultivation, highlighting the urgency of developing effective plant protection strategies.

Among the main fruit crops cultivated, the avocado tree (*P. americana* Mill) is particularly sensitive to thermal stress, making it one of the crops most affected by high temperatures and intense solar radiation. Sunburns caused by excessive temperatures and ultraviolet (UV) light can severely damage the leaves and fruits of avocado trees, resulting in a significant drop in fruit productivity and commercial quality [30]. These burns typically occur on the south and southwest sides of the trees, where solar radiation exposure is more intense and prolonged, causing fruit skin discoloration, which can progress to necrotic lesions, compromising both the commercial value and appearance of the fruits [11,31].

The avocado tree is particularly vulnerable to thermal stress, especially during flowering and fruiting stages. During flowering, high temperatures can result in a low fruit set rate due to increased floral abscission. This effect is exacerbated when high temperatures are combined with water stress, which limits the water availability necessary for healthy fruit development [26]. Additionally, intense heat can lead to rapid dehydration of plant tissues, causing cellular collapse and necrosis of young fruits, resulting in a sharp drop in avocado production and quality [32].

The sensitivity of the avocado tree to thermal stress is also related to its inadequate response to prolonged periods of intense solar radiation. Leaves and fruits exposed to direct sunlight for extended periods may suffer irreversible damage, such as severe burns and discoloration, which reduce the commercial value of the fruits. These lesions, caused by tissue overheating, not only affect the external appearance of avocados but also compromise internal integrity, reducing the post-harvest shelf life of the fruits. The avocado tree’s inability to quickly adapt to thermal stress conditions intensifies the need for management strategies to protect the plants and ensure the economic viability of production [11].

Thermal stress significantly affects the physiological processes of the avocado tree, especially photosynthesis and gas exchange [33]. Under high-temperature conditions, plants tend to close their stomata to minimize water loss through transpiration. However, this stomatal closure also reduces carbon dioxide (CO_2_) uptake, limiting the plant’s photosynthetic capacity. With less available CO_2_, glucose production rates decrease, hindering fruit growth and development. Additionally, thermal stress can lead to the accumulation of reactive oxygen species (ROS) in plant cells, causing oxidative damage that affects the functionality of organelles, such as chloroplasts, essential for photosynthesis [34].

To cope with thermal stress, the avocado tree activates a series of stress enzymes, such as superoxide dismutases (SOD), catalases (CAT), and peroxidases (POD), which detoxify free radicals accumulated during periods of excessive heat. However, the efficiency of these enzymes may be limited under prolonged or severe stress conditions, leading to the accumulation of oxidative damage. Furthermore, increased temperatures can alter plant enzyme activity, destabilizing metabolic processes and resulting in lower efficiency in nutrient assimilation and energy production. These physiological changes directly affect the health and productivity of the avocado tree, making it even more susceptible to damage caused by thermal stress [34].

The consequences of thermal stress on avocado fruit quality are significant, affecting sensory and nutritional attributes that determine the commercial value of production. High temperatures during fruit development can result in firmer or fibrous textures and cause undesirable changes in color and flavor, such as the development of brown tones or bitter flavors. The nutritional composition of the fruits is also compromised, with reduced levels of sugars, vitamins, and phenolic compounds, which are essential for nutritional and organoleptic quality. These impacts not only compromise the acceptance of the fruits in the market but also directly affect producers’ profitability [31].

Managing thermal stress in fruit trees, especially in sensitive crops like avocado, represents a significant agronomic challenge. The lack of adequate agronomic practices to protect plants during critical development stages, such as flowering and fruiting, can result in considerable losses. Additionally, the absence of precise definitions regarding the dosages and optimal timing for sunscreen application hinders the implementation of effective strategies. Producers often face difficulties balancing the need for plant protection with the ease of removing the applied products, especially in crops destined for export, where fruit appearance is a determining factor for market acceptance [33].

### 3.3. Sun Protection Strategies in Avocado Trees

#### 3.3.1. Kaolin

Sun protection strategies in avocado trees have proven essential for managing thermal stress. Among the most studied methods is the use of kaolin, due to its favorable physicochemical properties. Kaolin, when applied to plants, forms a physical barrier that reflects solar radiation, reducing the temperature of the leaf and fruit surfaces. Additionally, it is a non-expansive, non-abrasive material that easily disperses in water and does not chemically react over a wide pH range. According to the Environmental Protection Agency (EPA), kaolin is classified as a low-risk pesticide due to its low toxicity properties for humans and non-target organisms, as well as its lack of contribution to groundwater contamination or runoff [35].

Some kaolin-based products, applied via spraying, have demonstrated that the reflectivity of the plant surface can mitigate abiotic stress [15], primarily by reducing the temperature of leaves and fruits. Ref. [34] inferred that such products could lower leaf temperature by increasing reflectance and reducing transpiration. Its application also minimizes water loss through transpiration, contributing to plant health maintenance under thermal stress conditions. Moreover, kaolin is easy to apply using conventional sprayers, and its chemical composition does not significantly interfere with plant physiology, making it a viable and safe option for managing thermal stress in avocado trees.

Kaolin, applied to leaves and fruits in concentrations of 3 to 5%, helps reduce temperature and water loss from these structures [36]. However, when avocado fruits reach packing houses, removing this protector can be challenging, affecting the fruit’s market acceptance [33]. For easier recommendations to producers, it is important that, in addition to protecting the plants, these products can also be easily applied with traditional sprayers and removed from the fruit surface after harvest.

Besides kaolin, other sun protection products have been compared in recent studies, including titanium dioxide and calcium oxide. Titanium dioxide, known for its high capacity to reflect ultraviolet light, provides additional protection against UV radiation damage and helps reduce leaf temperature. Calcium oxide, applied in suspension form, acts similarly to kaolin, creating a protective layer that reduces direct solar radiation incidence on the plant. These products were tested at different stages of plant development, from flowering to fruiting, proving effective in reducing damage caused by thermal stress.

**Figure 1:**
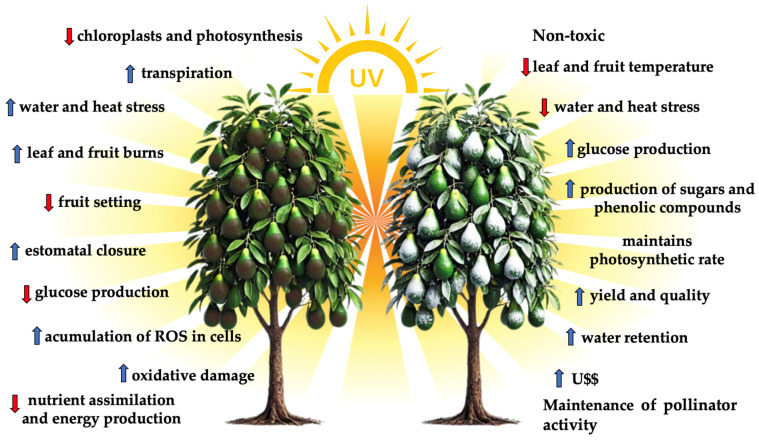
Graphical abstract of sun protection as a strategy for managing heat stress in avocado trees, 2024.

Choosing the most suitable product for use depends on several factors, including the cultivated species, environmental conditions, the plant’s development stage, and the ease of applying and removing the products after harvest. Specific case studies on avocado trees, such as those conducted by [36]. showed that applying kaolin at concentrations of 3% to 5% during periods of high solar intensity resulted in a significant reduction in leaf temperature and improved water retention in plants. This, in turn, led to increased production of high-quality fruits with a lower incidence of sunburn (Figure 1).

However, one of the challenges associated with using kaolin and other sunscreens is removing product residues during the post-harvest process. In cases where the product is not easily removed, there may be a reduction in market acceptance, especially in international markets where fruit appearance is a crucial quality criterion [33].

The application methods for these sunscreens vary according to the type of product and the specific needs of the crop. Foliar sprays with kaolin and titanium dioxide are commonly used as they allow uniform coverage of leaves and fruits, ensuring effective protection against thermal stress. The concentrations of the applied products can vary between 2% and 8%, depending on environmental conditions and the plant’s development stage. In fruit trees such as avocado, citrus, grapevines, and apple trees, the application of these products has demonstrated a significant reduction in leaf and fruit temperature, as well as an improvement in the quality of harvested fruits. However, to ensure the fruits’ efficiency and commercial acceptance, it is essential that the products are easily removed during the post-harvest process, especially in crops destined for export. This factor is particularly relevant for avocados, whose exports have increased significantly in recent years, driven by the global demand for healthy and nutrient-rich foods [17].

#### 3.3.2. Films and Shading Screens 

The use of films and shading screens has also been explored in other fruit crops, with the latter being particularly effective in intensive cultivation systems where solar radiation can be more precisely controlled. Ref. [37], evaluating the potential of white shading screens with 25% solar radiation retention in orange crops, inferred a positive effect on thermal stress protection in plants. Covering ‘Honeycrisp’ apple trees in pots under thermal stress and high light conditions with blue shading screens with 22% radiation retention improved the efficiency of photosynthetic light use at the leaf level and reduced photoinhibition symptoms [38].

The use of silver shading screens at 60% can significantly reduce thermal stress in ‘Pinkerton’ avocado trees during extreme heat events [30]. According to [39], seaweed extracts can also be used to protect plants against abiotic stresses, as their application is associated with increased plant tolerance to such adversities.

#### 3.3.3. Seaweed Extracts

Improvements in applying seaweed extracts, such as *Kappaphycus alvarezii*, are related to regulating stress-associated genes, defense, phytohormones, nitrogen metabolism, signal transduction, photosynthesis, ion transport, antioxidant pathways, and polysaccharide metabolism in plants. Additionally, this extract demonstrates antibacterial and antifungal properties, resulting in better quality and prolonged fruit shelf life [40]. Furthermore, the application of *K. alvarezii* can mitigate the impact of climate change by up to 11.4%, as indicated by [40].

Despite the evident benefits, the implementation of sun protection strategies in avocado trees still faces challenges. One such challenge is the variation in product effectiveness depending on climatic conditions and the cultivated species. Furthermore, there is a continuous need for research to optimize doses and application methods to maximize plant protection without compromising fruit quality. Integrating these practices with other management techniques, such as controlled irrigation and the use of heat-resistant cultivars, is also essential to ensure the sustainability of avocado production in regions prone to thermal stress [41].

### 3.4. Impacts of Sunscreens on Avocado Production and Quality

Sunscreens have been widely researched in avocado trees, with several case studies documenting their positive effects. In particular, the application of products such as kaolin has been the subject of numerous studies due to its ability to reflect solar radiation and thus reduce the temperature of leaf and fruit surfaces. These studies show that the use of kaolin, especially during periods of high solar radiation, significantly contributes to protecting plants against damage caused by thermal stress, such as sunburn, which is common in hot climates [36].

Experimental results have highlighted that applying sunscreens to avocado trees not only reduces leaf temperature but also helps maintain the photosynthetic rate, which is essential for healthy plant growth and fruit development. The prevention of sunburn has a direct impact on fruit quality, resulting in avocados with a more uniform texture, better coloration, and nutritional composition. These improvements in fruit quality are especially important for export markets, where appearance and sensory quality are critical criteria for product acceptance [41].

Furthermore, applying sunscreens can improve the water retention of plants, which is crucial during drought periods or under high-temperature conditions. Maintaining an adequate hydration level in leaves and fruits helps reduce water stress, contributing to better performance of avocado trees. In practical terms, producers who adopt the use of sunscreens often report increased productivity, with a higher proportion of fruits meeting the quality standards required for commercialization in both domestic and international markets. This can result in a more favorable economic return, offsetting the initial costs of applying the products.

However, the use of sunscreens in avocado trees also requires careful consideration regarding the removal of residues from the fruits after harvest. Residues from products like kaolin can remain on the fruit surface, negatively affecting their appearance and consequently their marketability, especially in markets that value the immaculate appearance of products. To mitigate this challenge, it is crucial that post-harvest procedures include effective techniques for removing sunscreens, ensuring that the fruits are visually appealing and comply with the quality standards required by consumers. Practices such as washing the fruits with specific solutions or using suitable machinery to remove residues may be necessary to ensure that the fruits are accepted in the target markets.

Beyond the technical aspects, the costs associated with the application and removal of these products must be considered by producers. The economic viability of these practices on a large scale can vary depending on the operation size and target market. For example, while large producers may justify the investment in application and removal technology, small producers may need additional support, such as subsidies or technical training, to effectively adopt these practices. Rural extension programs and cooperation among producers can play an important role in disseminating these technologies and adapting them to local conditions [33].

Finally, it is important for producers to continuously monitor the effectiveness of sunscreens, adjusting management practices as necessary to optimize results. This includes evaluating specific climatic conditions, choosing the ideal time for application, and possibly integrating other management strategies, such as controlled irrigation and the use of more heat-resistant cultivars. By adopting an integrated approach, producers can maximize the benefits of using sunscreens and ensure the production of high-quality avocados, even under challenging climatic conditions.

### 3.5. Challenges and Opportunities in the Use of Sunscreens in Avocado Trees

The use of sunscreens in avocado trees, despite their proven benefits, faces some practical limitations that need to be addressed to maximize their effectiveness. One of the main current limitations is the precise definition of dosages and the ideal timing for application. The variability in environmental conditions, such as temperature, humidity, and solar radiation intensity, can significantly influence the effectiveness of sunscreens. Studies indicate that excessive application may not only be inefficient but also result in unnecessary costs, while insufficient application may not provide adequate protection. Moreover, the timing of application is crucial, as the effectiveness of sunscreens can be maximized if applied during times of the day with the highest risk of exposure to intense solar radiation [36].

Another challenge associated with using sunscreens is the potential negative impact on the commercial acceptance of the fruits. As mentioned earlier, visible residues of sunscreens, such as kaolin, may remain on the fruit surface after harvest, negatively affecting their appearance and, consequently, their acceptance in markets, especially in export markets, which are highly demanding in terms of visual quality. This risk must be mitigated through effective post-harvest techniques that ensure the complete removal of residues without compromising fruit integrity. Additionally, producers should consider the economic impact of these practices, evaluating whether the benefits of using sunscreens outweigh the costs associated with application and removal [33].

Given these challenges, there is a clear need to develop specific agronomic practices tailored to local conditions and the needs of producers. This includes creating more precise application protocols that consider environmental variables and the specific characteristics of avocado cultivation. Moreover, it is essential to provide technical training to fruit growers, enabling them to properly apply sunscreens and manage crops under thermal stress conditions. Rural extension programs and cooperation initiatives among producers, researchers, and input supplier companies can play a crucial role in disseminating these practices and improving management techniques, ensuring that fruit growers can fully benefit from the advantages offered by sunscreens [41].

While the challenges are significant, the opportunities for adopting sunscreens in avocado trees are also promising. With the increasing demand for high-quality fruits, both in domestic and international markets, the effective implementation of these practices can provide a competitive edge to producers. The combination of sunscreens with other management technologies, such as precision irrigation and the use of more heat-resistant cultivars, can help ensure the sustainability of production in the face of climate change and adverse growing conditions [42,43]. Therefore, investment in research, development, and training is essential for these opportunities to be fully realized.

### 3.6. Future Directions for Research and Development

Despite significant advances in the use of sunscreens in fruit trees, considerable gaps in research still need to be addressed to optimize these practices. One of the main challenges is the need for more in-depth studies that consider regional climatic variability and the specific responses of different avocado cultivars to sunscreens. Additionally, the interaction between sunscreens and other management factors, such as irrigation and nutrition, remains underexplored, leaving a gap in understanding how these products can be more effectively integrated into daily agricultural practices [36].

To fill these gaps, it is essential for future research to focus on evaluating new sunscreen formulations that can offer more durable and effective protection under different environmental conditions. This includes developing products that are easier to apply and remove and have less visual impact on the fruits, thereby ensuring broader commercial acceptance. Research should also investigate the combination of sunscreens with other technologies, such as climate sensors and real-time monitoring systems, which can optimize the timing of application and maximize treatment efficiency [41].

The successful adoption of these technologies in the field heavily depends on the training of producers and the support offered by rural extension programs. The continuous education of fruit growers in integrated management practices, including the proper use of sunscreens, is crucial to ensure that new technologies are applied effectively and sustainably. Rural extension programs play a fundamental role in disseminating knowledge and adapting practices to local realities, enabling producers not only to improve the quality of their products but also to increase their competitiveness in the global market [42,43].

While there are promising indications, it is important to consider that the response to the use of sunscreens in avocado trees still depends on environmental conditions and the type of sunscreen used. However, for these techniques to be successful, strategies aimed at using these technologies in partnership with research institutions and producers in different producing regions are necessary. In summary, while the use of sunscreens in avocado trees already shows clear benefits, future directions for research and development should focus on optimizing these practices and overcoming current challenges. Collaboration among researchers, producers, and rural extension agents is essential to promote innovations that meet the needs of fruit growers, ensuring the sustainability and productivity of crops in a scenario of increasing climate change.

## 4. Conclusions

Sun protection has proven to be an effective strategy in managing thermal stress in avocado trees, offering a practical solution to mitigate the adverse impacts of intense solar radiation and high temperatures. The application of sunscreens, such as kaolin, has been extensively studied and shows significant benefits, including reducing leaf temperature and preventing sunburn, which helps maintain fruit quality and plant productivity. However, challenges remain, such as the need to define appropriate dosages and optimal application times, as well as dealing with residues that may compromise fruit appearance, especially for demanding markets.

The continuous development of new formulations and the integration of these practices with other technologies, such as precision irrigation and the use of heat-resistant cultivars, are crucial for optimizing the use of sunscreens. Additionally, training producers and supporting rural extension programs are essential to ensure the successful adoption of these technologies in the field.

In a scenario of increasing climate change, implementing sun protection strategies not only helps sustain avocado production but also provides a competitive advantage in the global market. Collaboration among researchers, producers, and rural extension agents will be crucial to overcoming current challenges and promoting innovations that ensure the economic viability and sustainability of avocado cultivation in Brazil.

## Data Availability

Not applicable.
